# Left Transradial Neurointervention Using a 3-French Simmons Guiding Sheath for a Left Carotid Approach in Patients With an Aberrant Right Subclavian Artery: A Technical Note on a Case of Preoperative Embolization of Intracranial Meningioma

**DOI:** 10.7759/cureus.77944

**Published:** 2025-01-24

**Authors:** Taigen Sase, Hidemichi Ito, Kiyotaka Wakatsuki, Homare Nakamura, Hidetoshi Murata

**Affiliations:** 1 Department of Neurosurgery, St. Marianna University School of Medicine, Yokohama Seibu Hospital, Yokohama, JPN; 2 Department of Neurosurgery, St. Marianna University School of Medicine, Kawasaki, JPN

**Keywords:** arterial lusoria, left radial access, meningioma, transradial neurointervention, tumor feeding embolization

## Abstract

An aberrant right subclavian artery (ARSA) is a rare variant of the normal aortic arch anatomy. Right transradial carotid artery cannulation is extremely challenging in patients with ARSA. Herein, we present a case of a right falcine meningioma with an ARSA that was successfully accessed with a 3-French Simmons guiding sheath via the left transradial approach. Additionally, preoperative embolization of the feeding middle meningeal artery (MMA) was performed. Here, we report our surgical technique. An 80-year-old woman was diagnosed with a right falcine meningioma with ARSA. The meningioma exhibited tumor staining in the parietal branch of the left MMA. We planned a preoperative MMA embolization via the left radial artery. After the 3-French Simmons guiding sheath was engaged in the left common carotid artery (CCA) using the pull-back technique, a triaxial system (3-French Simmons guiding sheath/3.2-French distal access catheter/microcatheter) was implemented. The 3-French guiding sheath to the left CCA was successfully achieved using the pull-back technique. Distal access catheter guidance to the proximal left MMA was successfully achieved without catheter kinking or systemic instability. However, guiding the microcatheter beyond the pterional segment of the left MMA parietal branch because of the severe curvature and tortuosity of the vessel was difficult. Thus, embolization with liquid and particulate embolic materials was abandoned, and tumor flow reduction was performed using coil embolization of the MMA. Three days after the neurointervention, craniotomy tumor removal was successfully performed achieving near-total resection of the tumor. Thereafter, no radial artery occlusion was observed at the puncture site. The patient was discharged from our hospital two weeks after craniotomy surgery. The left transradial artery approach using a 3-French Simmons guiding sheath is useful for left carotid artery cannulation in patients with ARSA.

## Introduction

The aberrant right subclavian artery (ARSA) is a rare variant in which the right subclavian artery originates abnormally. The ARSA is defined as a right subclavian artery originating directly from the aortic arch and distal to the origin of the left subclavian artery [1]. A prevalence of 0.6%-1.4% of ARSA has been reported [2].

Historically, neurointerventions have been performed using a transfemoral approach (TFA). However, coronary intervention studies have demonstrated the apparent benefits of the transradial approach (TRA), including a low risk of bleeding and vascular complications, shortened recovery time, and early postprocedural ambulation [3,4]. Transradial neurointervention (TRN) has become increasingly popular. Nevertheless, in patients with ARSA, carotid artery cannulation via the right radial artery is technically challenging due to the extremely undesirable trajectory [5,6].

The 3-French Simmons guiding sheath is a radial-specific neurointerventional sheathless guide catheter with a kink-resistant “pre-shaped” Simmons curve at the distal end, which is specifically designed for common carotid artery (CCA) cannulation. Herein, we present a case of a right falcine meningioma with an ARSA that was successfully accessed with a 3-French Simmons guiding sheath via the left radial artery, and preoperative embolization of the feeding middle meningeal artery (MMA) was performed. We report an endovascular surgical technique.

## Technical report

**Figure 1:**
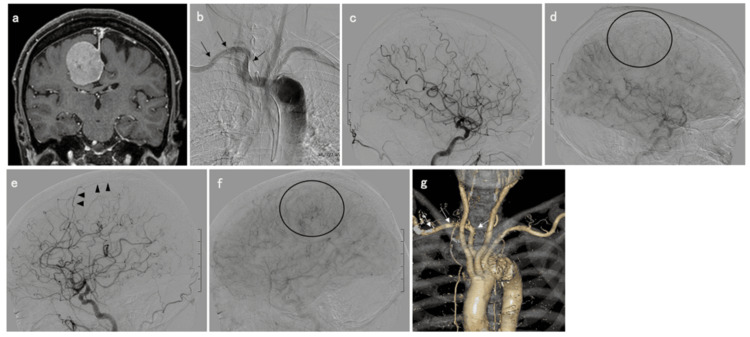
Preoperative MRI and angiography a: Contrast-enhanced MRI displaying a right falcine meningioma with a maximum diameter of 40.1 mm. b: Aortic arch angiography through the catheter confirmed an ARSA (black arrows). c, d: Right common carotid artery angiography exhibiting no tumor staining (black circle) of the meningioma (b: early arterial phase and c: delayed arterial phase). e, f: Left common carotid artery angiography (d: early arterial phase, e: delayed arterial phase) demonstrating meningioma stain (black circle) through the middle meningeal artery parietal branch (black arrowheads). g: Aortic arch contrast computed tomography angiography revealing ARSA (white arrows). MRI, magnetic resonance imaging; ARSA, aberrant right subclavian artery.

An 80-year-old right-handed woman was diagnosed with a brain tumor at another hospital after undergoing a plain magnetic resonance imaging (MRI) scan to determine the cause of her dizziness. She was referred to our hospital for the treatment of a brain tumor. Her dizziness improved, but she had slight left lower limb paralysis. Contrast MRI revealed a right falcine meningioma with a maximum diameter of 40.1 mm (Figure 1a). To confirm the artery feeding the tumor, cerebral angiography was performed using the right transbrachial approach. Guiding a 4-French Simmons diagnostic catheter into the right brachiocephalic artery and left CCA was challenging. Aortic arch angiography through the catheter confirmed ARSA (Figure 1b). The guidewire was directed at the aortic valve, and only the tip of the catheter was guided to the right and left CCAs. Angiography revealed that the main feeding artery was the left MMA parietal branch (Figure 1c-1f). Cerebral angiography was performed without any complications. Subsequently, the ARSA was confirmed using aortic arch contrast computed tomography (CT) angiography (Figure 1g). Preoperative MMA embolization and craniotomy tumor removal were planned for symptomatic meningiomas after explaining the procedures to the patient. We planned a preoperative MMA embolization via the left radial artery.

**Figure 2:**
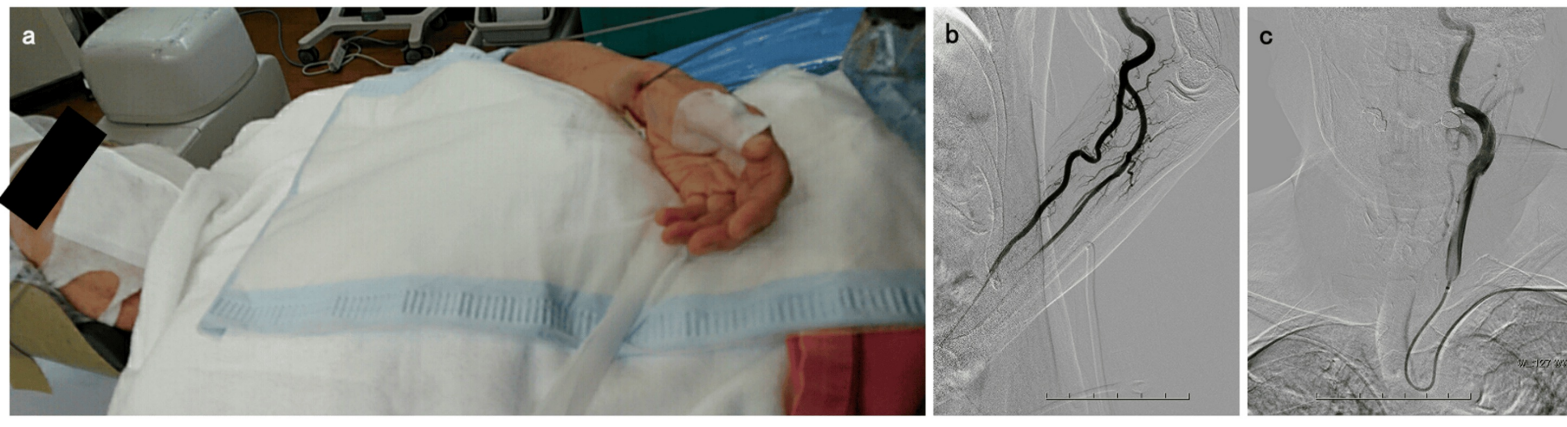
The positioning and procedure for introducing the guiding sheath in an endovascular procedure a: The left forearm placed in the sheath is fixed to the patient's lower abdomen in a position with minimal strain. b: Angiography is performed through the sheath of the left radial artery. c: The 3-French Simmons guiding sheath is introduced into the left common carotid artery using the pull-back technique.

**Figure 3:**
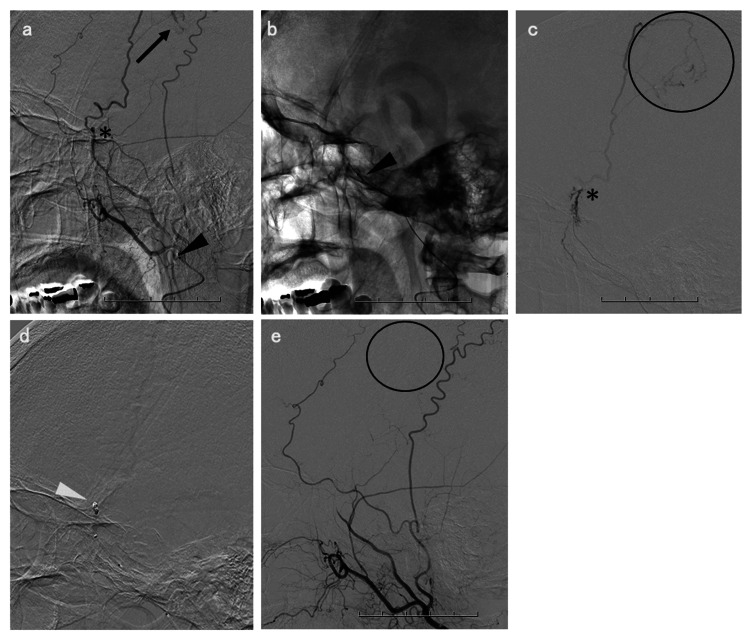
Embolization procedure a: Distal access catheter is guided to the left external carotid artery using the guidewire under roadmap guidance. Angiography, performed through the distal access catheter, demonstrates the meningioma stain (black arrow) through a middle meningeal artery parietal branch. In addition, curvature and tortuosity are detected in the pterional segment of the left MMA parietal branch. b: The distal access catheter is guided further distally to target the MMA temporal segment, along the axis of the microguidewire and the microcatheter. The black arrowhead represents the distal end of the distal access catheter. c: Angiography performed through the distal access catheter displaying the tumor stain (black circles). d: Coil embolization (white arrowhead) is performed. e: Postembolization left external carotid artery angiography revealing the disappearance of the tumor stain (black circle).

**Figure 4:**
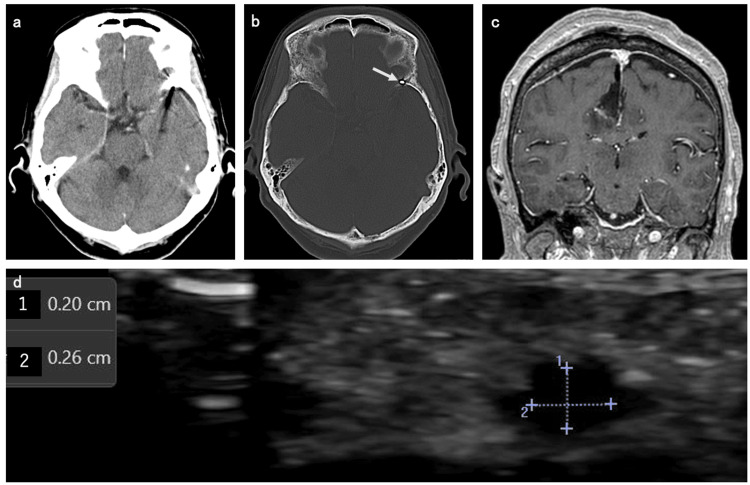
Postprocedural findings a, b: Postembolization computed tomography scans revealed no complications, including epidural hematoma, and embolized coils (white arrow). c: Postoperative contrast magnetic resonance imaging displays nearly total resection of the meningioma. d: No left radial artery occlusion at the puncture site confirmed using ultrasound examination.

Under local anesthesia and mild sedation, a 4-French short sheath was introduced into the classical left radial artery. The left forearm in which the sheath was placed was fixed to the patient's lower abdomen in a position that placed minimal strain (Figure 2a). After the intra-arterial injection of nitroglycerin and heparin sodium, angiography was performed through the sheath to confirm the brachial artery along with its bifurcation (Figure 2b). Under roadmap guidance, the short sheath was replaced with a 3-French Simmons guiding sheath (outer diameter, 5.3-French, 0.070 inch, 1.76 mm; inner diameter, 4.5-French, 0.059 inch, 1.5 mm; usable length, 93 cm; 3F Axcelgude Stiff-J; Medikit, Tokyo, Japan), using a 0.035-inch Radifocus Guidewire (180 cm; Terumo, Co., Tokyo, Japan). Subsequently, the guidewire and 4-French coaxial catheter (usable length, 130 cm; 3-French inner catheter SY2; Medikit, Tokyo, Japan) were guided into the descending aorta. Using the descending aorta anchoring technique with the guidewire and catheter as described above, the distal end of the 3-French Simmons guiding sheath was reformed to the natural Simmons curve within the ascending aorta. A 3-French Simmons guiding sheath was introduced into the left CCA using the pull-back technique without any complication (Figure 2c). Following left CCA angiography, a 3.2/3.4-French distal access catheter (usable length, 120 cm; Guidepost; Tokai Medical Products, Inc., Aichi, Japan) was guided to the left external carotid artery using a guidewire under roadmap guidance (Figure 3a). As an additional microcatheter, an Excelsior SL-10 microcatheter (usable length, 150 cm; Stryker Neurovascular, Fremont, CA) was engaged into the MMA beyond the foramen spinosum, using 0.014-inch microguidewire (200 cm; ASAHI CHIKAI; Asahi Intecc, Co., Aichi, Japan), after the guidewire was removed. The distal access catheter was advanced further distally, targeting the distal MMA temporal segment, along the axis of the microguidewire and microcatheter (Figure 3b). Using a left TRA, distal access catheter guidance was successfully achieved without catheter kinking or system instability. Angiography was performed through the distal access catheter along with tumor staining (Figure 3c). However, guiding the microcatheter using a microguidewire beyond the pterional segment of the left MMA parietal branch because of the severe curvature and tortuosity was challenging (Figure 3a, 3c). We observed a finding resembling an arteriovenous fistula, possibly due to manipulation of the microcatheter or microguidewire. Thus, embolization with embolic materials was abandoned, and feeding flow reduction for the tumor was performed using coil embolization of the MMA (Figure 3d). Left external carotid artery angiography revealed the disappearance of the tumor stain due to the left MMA parietal branch (Figure 3e). Postprocedural CT revealed coils placed at the MMA with no adverse events, including epidural hematoma (Figure 4a, 4b). No procedure-related or access-site complications were observed.

She was able to use her right and dominant hands immediately after the procedure. Three days after TRN, craniotomy tumor removal was performed, achieving nearly complete resection (Figure 4c). The amount of blood loss was 200 ml, and the surgical time was about 7 hours. Ten days after TRN, no radial artery occlusion at the puncture site was confirmed on ultrasound examination (Figure 4d). The patient was discharged from our hospital two weeks after craniotomy surgery.

## Discussion

ARSA is a rare variant of the right subclavian artery and is defined as a right subclavian artery originating directly from the aortic arch and distal to the origin of the left subclavian artery [1]. A prevalence of 0.6%-1.4% of ARSA has been reported [2]. An association between Down syndrome and ARSA has been suggested, with a prevalence of 26%-34% [2]. 

Historically, diagnostic cerebral angiography and neurointerventions have been performed using TFA. However, coronary intervention studies have demonstrated apparent benefits of TRA, including a reduced risk of bleeding and vascular complications, shortened recovery time, and early postprocedural ambulation [3,4]. In addition, there are fewer postoperative bed rest restrictions compared to TFA, which may help prevent cognitive decline and disuse syndrome in elderly patients. Recently, these advantages have promoted the TRN for cerebral aneurysm coiling, carotid artery stenting, and others [7-10]. Therefore, TRN has garnered significant popularity. Currently, we have adopted radial-first neurointervention. Moreover, we frequently perform “left” TRN [11,12]. One benefit of the left TRN is that right-handed patients can use their dominant hand immediately after the procedure, as in our presentation. Furthermore, the left TRA is particularly useful in ARSA.

In patients with ARSA, CCA cannulation via the right TRA is technically challenging attributed to extremely undesirable trajectory [5,6]. Despite these difficulties, cases have been reported where ARSA was accidentally identified during endovascular treatment for a cerebral aneurysm using TRA, but the procedures were continued successfully [13-15]. However, a right TRA may result in life-threatening ARSA dissection [16,17]. We continued diagnostic cerebral angiography via the right radial artery even after the ARSA was identified; however, in some cases, the procedure was discontinued due to the risk of ARSA dissection.

In our case, the classical left TRA was appropriately utilized for preoperative tumor embolization of the MMA feeders, as the ARSA was detected preoperatively. If a left TRA is selected, the distal TRA should be an option because it allows both the surgeon and the patient to perform the procedure in a natural position. Although catheter kinking has occasionally been reported, our recent studies reported that left TRN using a 6-French Simmons guiding sheath provided a high procedural success rate without catheter kinking or system instability [11,12]. Additionally, CCA cannulation via the left radial artery has been reported as a technically feasible alternative to TFA in patients with ARSA [18,19]. Left transradial CCA cannulation using the pull-back technique is technically simple and unique to the radial-specific Simmons guiding sheath. This method is carefully and thoroughly explained in the reports by Inomata et al. [18] and Ito et al. [11].

In our technique, a 3-French Simmons guiding sheath was employed via the left radial artery to perform endovascular treatment for tumor feeder embolization. This technique is summarized as follows: The Simmons curve was successfully reformed within the ascending aorta using the descending aorta anchoring technique. The Simmons guiding sheath was fully engaged in the left CCA using the pull-back method. Distal access catheter guidance to the proximal left MMA was successfully achieved without catheter kinking or system instability, owing to stable guiding sheath placement. Although the distal access catheter was guided to the distal temporal segment of the MMA, stability was maintained. However, guiding the microcatheter beyond the pterional segment of the left MMA parietal branch because of the severe curvature and tortuosity was difficult. This technique could be a viable therapeutic option for accessing extracranial arteries, including the MMA, because of the stability of the guiding sheath and distal access catheter through the left radial artery without catheter kinking.

In this study, we present a case of preoperative embolization of a meningioma in a patient with ARSA in whom left TRA using a 3-French Simmons guide sheath was a useful treatment option for left carotid artery cannulation.

## Conclusions

In patients with ARSA, the right TRA has been technically challenging attributed to an extremely undesirable trajectory. The left TRA using a 3-French Simmons guiding sheath might be one of the effective neurointerventions for left carotid artery cannulation, although preoperative detection of ARSA is required.
